# Annotations

**Published:** 1895-09-21

**Authors:** 


### ANNOTATIONS.

### Discredited Hospitals.

THE establishment of the so-called Jubilee Hospital at Brompton, an over-crowded tenement house, where people are wrongly taken in for medical treatment, and its continuance without the interference of the health authorities of the district, is a grave public scandal. They manage these things better in the United States. Last year a law was passed which enacted that all corporations established in New York State for the purpose of giving medical and surgical relief must have their certificates of incorporation approved by the Board of Charities before they can continue their work. This law renders it impossible for private persons to open such a building in America as that now designated the Jubilee Hospital and to treat patients in it. We have already called upon the medical officer of health for the Brompton district to inspect the Jubilee Hospital as he would inspect any other over-crowded insanitary tenement and to put the law in force. If the medical officer of health fails in his duty in this respect other proceedings may be necessary as a protection to the innocent members of the public whose health and lives may be imperilled by his neglect to discharge a plain duty entrusted to him by law. Our contemporary *Truth* has exposed the extraordinary character of the financial arrangements of this private venture hospital, in the same way that we have exposed its sanitary and medical abuses in THE HOSPITAL of August 10th. It constitutes a danger to the poor and to the deservedly high reputation of the great general hospitals of London. Matters cannot rest as they stand at present, and the scandal of the existence of the "Jubilee Hospital" cannot long be permitted to continue.

### Buried Alive.

THE old scare about being buried alive has recently cropped up again, and it is one so full of horror to all who look on burial as the proper end of earthly existence that we cannot wonder at the eagerness with which the subject is discussed whenever an ingenious writer puts it judiciously before the public—which, in truth, is not seldom. It would be idle to pretend that such an event as the burial of a person before life has ceased never happens, but the reports of such occurrences mostly come from abroad, where early burial is the custom, and where the temperature often prevents the corpses attaining that degree of coldness which with us is looked upon as, and is, in fact, so distinctive a sign of death. In England, however, things are different. Burial is usually considerably delayed, and in the vast majority of cases there is ample opportunity for certainty, and for the fact of death becoming obvious to the ordinary understanding. We believe that such an event as premature burial must be of the extremest rarity in this country. Nevertheless, death is not always regarded with due solemnity; we too often hear of levity and carelessness on the part of those whose duty it is to deal with the bodies of the dead, and in discussing this matter in all seriousness, as alone it should be discussed, it is no use shirking the fact that the Englishman's only protection from this terrible fate lies in the lapse of time before burial takes place, and in the exercise of common sense by a set of women who are not always to be relied upon. People lay great store upon certificates of death, forgetting—perhaps not knowing—that no professional certification or even ascertainment of the fact of death having taken place is required by law in England. The law imposes on every registered medical practitioner the duty of giving a certificate, stating to the best of his knowledge and belief the cause of the death of his patient, but it carefully avoids requiring that the fact of death should be either certified or ascertained, and no certificate even of the cause is required when the deceased has been attended by a quack, or by no one at all. In such cases the informant merely states the fact of the death having taken place, and says what he thinks was the nature of the disease, and, if there is nothing to arouse suspicion, the registrar writes "uncertified" opposite the entry and there is an end of the matter. The law then gives no protection against premature burial, for, unless a relative be present at the death, the informant, on the strength of whose statement the burial certificate is issued, may not have seen the patient after death, and possibly not even for some time before. It is a very haphazard proceeding, and no one can assert that death registration in England is on a satisfactory basis so long as the fact of death having taken place is allowed to hinge on the impressions of some ignorant old lady who has "laid out" the corpse, checked only by a momentary peep at the half-covered face by a few relatives, who may be either totally indifferent, or may be so upset by emotion that their inspection is worthless. It is not a satisfactory state of affairs.

### Polygamy and Progress.

A WRITER in *Harper's Magazine* gives a new explanation of the ill-feeling between Moslems and Christians, which has resulted in the Armenian massacres. With all the disadvantages under which they labour, the Christians in Armenia acquire flocks and herds, become prosperous, and outrun the followers of Islam. And this prosperity the American critic traces, in part at least, to monogamy. The Moslem wife has no secure position. There is no honest comradeship between them, and comradeship rather than passion is the basis of married life. Very different is the position of the Christian wife. While she lives it is secure, she has no rival, and her husband's profit is her gain also. Hence she can be his friend and fellow worker, knowing that all she can make or save is for herself and her children. And so, even in the most disadvantageous circumstances, the Christians flourish. One cannot but infer that elsewhere than in Armenia the solidarity of the family, only to be obtained by permanence of the marriage bond and by monogamy, is the foundation of sound and prosperous national life.

